# Extracellular mixed histones are neurotoxic and modulate select neuroimmune responses of glial cells

**DOI:** 10.1371/journal.pone.0298748

**Published:** 2024-04-17

**Authors:** Dylan E. Da Silva, Christy M. Richards, Seamus A. McRae, Ishvin Riar, Sijie (Shirley) Yang, Noah E. Zurfluh, Julien Gibon, Andis Klegeris

**Affiliations:** Department of Biology, University of British Columbia Okanagan Campus, University Way, Kelowna, British Columbia, Canada; University of Michigan Medical School, UNITED STATES

## Abstract

Although histone proteins are widely known for their intranuclear functions where they organize DNA, all five histone types can also be released into the extracellular space from damaged cells. Extracellular histones can interact with pattern recognition receptors of peripheral immune cells, including toll-like receptor 4 (TLR4), causing pro-inflammatory activation, which indicates they may act as damage-associated molecular patterns (DAMPs) in peripheral tissues. Very limited information is available about functions of extracellular histones in the central nervous system (CNS). To address this knowledge gap, we applied mixed histones (MH) to cultured cells modeling neurons, microglia, and astrocytes. Microglia are the professional CNS immunocytes, while astrocytes are the main support cells for neurons. Both these cell types are critical for neuroimmune responses and their dysregulated activity contributes to neurodegenerative diseases. We measured effects of extracellular MH on cell viability and select neuroimmune functions of microglia and astrocytes. MH were toxic to cultured primary murine neurons and also reduced viability of NSC-34 murine and SH-SY5Y human neuron-like cells in TLR4-dependent manner. MH did not affect the viability of resting or immune-stimulated BV-2 murine microglia or U118 MG human astrocytic cells. When applied to BV-2 cells, MH enhanced secretion of the potential neurotoxin glutamate, but did not modulate the release of nitric oxide (NO), tumor necrosis factor-α (TNF), C-X-C motif chemokine ligand 10 (CXCL10), or the overall cytotoxicity of lipopolysaccharide (LPS)- and/or interferon (IFN)-γ-stimulated BV-2 microglial cells towards NSC-34 neuron-like cells. We demonstrated, for the first time, that MH downregulated phagocytic activity of LPS-stimulated BV-2 microglia. However, MH also exhibited protective effect by ameliorating the cytotoxicity of LPS-stimulated U118 MG astrocytic cells towards SH-SY5Y neuron-like cells. Our data demonstrate extracellular MH could both damage neurons and alter neuroimmune functions of glial cells. These actions of MH could be targeted for treatment of neurodegenerative diseases.

## Introduction

Neurons and glia are two broad categories of the central nervous system (CNS) cell types. Microglia are the resident mononuclear phagocytes of the brain that orchestrate neuroimmune responses and perform phagocytosis to remove harmful substances and tissue debris [1–3]. Astrocytes are another glial cell type, which are commonly viewed as the main support cells of neurons, but they also participate in neuroimmune processes. Both microglia and astrocytes respond to various insults, including exogenous pathogens, as well as endogenous pathology-associated structures, such as misfolded proteins, and damage-associated molecular patterns (DAMPs) [4, 5]. The latter group consists of very heterogenous molecules that are normally located intracellularly, but can be released from activated, damaged, or dying cells [5–7]. Examples of DAMPs include ATP, mitochondrial DNA, high-mobility group box (HMGB) 1, mitochondrial transcription factor A (TFAM), and cytochrome C [8–12]. After being released into the intercellular space, all these molecules can interact with the pattern recognition receptors (PRRs), such as toll-like receptors (TLRs), that are expressed by numerous cell types in both the periphery and the CNS [13–15].

DAMPs interact with the PRRs expressed by most peripheral immune cells causing their activation [16, 17]. Additionally, several DAMPs, including HMGB1 [18], cytochrome C [19], and TFAM [11] have been shown to induce a reactive state in microglia, the resident CNS immunocyte [20, 21], characterized by the upregulated secretion of several pro-inflammatory and potentially neurotoxic molecules, including tumor necrosis factor-α (TNF), C-X-C motif chemokine ligand 10 (CXCL10), reactive oxygen species (ROS), and the excitatory neurotransmitter glutamate [22–24]. In acute processes, including infection or trauma, the secretion of such molecules is important for cell-to-cell signalling, elimination of CNS pathogens, and the resolution of inflammation [25]. However, persistent death of neurons and their release of multiple DAMPs, such as ATP, HMGB1, TFAM, and cytochrome C, can cause sustained microglia reactivity, including their dysregulated phagocytic activity, contributing to chronic neuroinflammation [4, 6]. Protracted microglial reactivity is believed to be damaging or toxic towards other CNS cells and may further promote the neuronal death observed in Alzheimer’s disease (AD) and other neurodegenerative diseases [7, 26, 27]. Notably, elevated levels of the intracellular protein HMGB1 have been reported in the extracellular compartment of AD brains, which could be a result of increased death of neurons and may contribute to chronic activation of microglia [5]. Additionally, some DAMPs are directly toxic to cells. For example, extracellular ATP induces the death of HK2 human renal tubular epithelial cells and NG108-15 murine neuron-like cells [28, 29].

Prolonged exposure to DAMPs, such as HMGB1, cytochrome C, and TFAM, also dysregulates the immune responses of astrocytes and promotes their reactive phenotype [12, 30, 31]. Reactive astrocytes are characterized by increased expression of glial fibrillary acidic protein and upregulated secretion of pro-inflammatory and potentially neurotoxic molecules, such as ROS, glutamate, and matrix metalloproteinase-9 [32, 33]. Similar to microglia, astrocyte reactivity can lead to neuronal death and may contribute to the pathogenesis of neurodegenerative diseases (reviewed in [34, 35]). All these neuropathologies currently lack effective treatment options; therefore, targeting DAMPs, and the cellular receptors they interact with, has recently been considered as a potential strategy for abrogating glia-mediated immune responses and neurotoxicity in neurodegenerative disease. Studies on DAMPs of the CNS are currently lagging behind similar research with their counterparts in the peripheral tissues. Thus, the identification and characterization of novel DAMPs of the CNS could facilitate the discovery of new biological targets for treatment of AD and other neurodegenerative disorders.

Histones are intranuclear proteins involved in the spatial organization of DNA. The four core histones (H2A, H2B, H3, and H4) form the nucleosome complex, which DNA wraps around, while the linker histone (H1) associates with the linear DNA just outside the nucleosomes [36]. Extracellular histones are established DAMPs of the peripheral tissues where individual histone isoforms, as well as preparations of mixed histones (MH) containing all histone isoforms, have been shown to interact with PRRs, such as TLR4, of immune cells, inducing their pro-inflammatory responses [37]. For example, MH upregulate IL-6 production by primary human leukocytes *in vitro*, which is ameliorated by pre-incubation with anti-TLR4 antibodies [38]. Similarly, intravenous infusion of MH increases the serum level of this pro-inflammatory cytokine in wild-type, but not TLR4 knockout mice [39]. The latter study also demonstrates TLR4-dependent upregulation of peripheral TNF levels in response to MH infusion.

Very limited studies have explored the DAMP-like activity of extracellular histones in the context of the CNS pathophysiological processes. Both protective and toxic actions of extracellular MH towards neuronal cells have been reported [40–42]. To date, only three studies have investigated the effects of individual histone isoforms on microglia-like cells by measuring their expression of inflammatory markers and cytokines [39, 43, 44]. To the best of our knowledge, the effects of extracellular histones on microglial phagocytic activity or any of the immunomodulatory functions of astrocytes have not been reported. To address these knowledge gaps, we studied the actions of extracellular histones on several different cell lines modeling neurons, microglia, and astrocytes. Since cell activation and death likely lead to the simultaneous release of all histone isoforms [45], we used MH instead of individual histone types to examine their effects on viability of neuron-like and glial cells. In addition to monitoring MH-induced release of specific inflammatory mediators by microglia-like cells, we also studied the ability of MH to modulate the overall cytotoxic potential of glial cells. Extracellular histones have been reported to have not only harmful but also protective activity [46]; therefore, we added MH to both resting and immune-activated glial cells to investigate their potential neuroimmunomodulatory function. Moreover, to determine whether the neurotoxic action of MH observed in this study is mediate by TLR4, we conducted further studies with a selective TLR4 inhibitor, TAK-242.

## Materials and methods

### Reagents

MH from calf thymus (H9250-100MG), LPS from *Escherichia coli* O55:B5, the TLR4 inhibitor ethyl-(6*R*)-6-(*N*-(2-chloro-4-fluorophenyl)sulfamoyl)cyclohex-1-ene-1-carboxylate (TAK-242), 3-(4,5-dimethyl-3-thiazoyl)-2,5-diphenyl-2H-tetrazolium bromide (MTT), bisbenzimide (Hoechst 33258), *N*-(1-naphthyl)ethylenediamine dihydrochloride, sulfanilamide, dimethyl sulfoxide (DMSO), beta-nicotinamide adenine dinucleotide (β-NAD), triethanolamine, diaphorase from *Clostridium kluyveri*, L-glutamic acid monosodium salt, and *N*,*N*-dimethylformamide (DMF) were purchased from Sigma-Aldrich (Oakville, ON, Canada). Human and murine recombinant interferon (IFN)-γ, and enzyme-linked immunosorbent assay (ELISA) development kits for human TNF, and both human and murine CXCL10 were acquired from Peprotech (Embrun, ON, Canada). Fluorescein isothiocyanate (FITC) surface-labelled fluorescent polystyrene latex beads (one μm diameter) were purchased from Bangs Laboratories (Fishers, IN, USA). Iodonitrotetrazolium chloride (INT), and L-glutamic dehydrogenase were purchased from Cedarlane (Burlington, ON, Canada). Neurobasal medium and B27 supplement were purchased from Invitrogen (Burlington, ON, Canada). Calf bovine serum (CBS), Dulbecco’s modified Eagle medium nutrient mixture F-12 Ham (DMEM-F12), 0.05% and 0.25% trypsin with ethylenediaminetetraacetic acid (EDTA), penicillin/streptomycin/amphotericin B stock solutions, and all other reagents were obtained from ThermoFisher Scientific (Ottawa, ON, Canada).

### Cell cultures

The BV-2 murine microglia were donated by Dr. G. Garden (Department of Neurology, University of Washington, Seattle, WA, USA). The NSC-34 murine neuron-like cells were obtained from Dr. A. Milnerwood (Brain Research Centre, UBC, Vancouver, BC, Canada). The glioma-derived U118 MG human astrocytic cell line was purchased from the American Type Culture Collection (ATCC, Manassas, VA, USA). The SH-SY5Y human neuroblastoma cells were provided by Dr. R. Ross (Fordham University, Bronx, NY, USA). Cells were cultured in T75 flasks kept at 37 ˚C in a humidified 95% air and 5% CO_2_ atmosphere. All cultures were maintained in DMEM-F12 supplemented with 10% CBS, 100 U/ml penicillin, 100 μg/ml streptomycin, and 500 ng/mL amphotericin B. The same medium supplemented with 5% CBS and antibiotics was used for all cell culture experiments described below except for toxicity studies with primary murine neurons, which were incubated in Neurobasal medium.

Primary cultures of cortical neurons were prepared from C57BL/6NCrl mice (Charles River Laboratories, Canada) as previously described with minor modifications [47]. All experimental procedures involving animals (protocol A20-0105) were in compliance with the guidelines of the Canadian Council on Animal Care and were approved by the research ethics committee of our university: The University of British Columbia Animal Care Committee, Vancouver, BC, Canada. Animals were housed under standard conditions with a 12 h light/dark cycle and had *ad libitum* access to water and food. Timed-pregnant (embryonic day 13) female mice were intraperitoneally injected with tribromoethanol (250 mg/kg) and returned to their cages in low-light conditions to minimize stress and aid anesthesia induction. Tribromoethanol (also known as Avertin) anesthesia followed by cervical dislocation is a rapid alternative to CO_2_/isoflurane that avoids hypoxia induced by CO_2_ as well as the neuroprotective effects of isoflurane. After 10–15 min, and in the absence of corneal and pedal withdrawal reflexes, cervical dislocation was performed, uterus dissected from the abdominal cavity, and embryos immediately decapitated using sharp scissors.

Cortical tissues from embryonic day 13 mice of either sex were dissected in cold Hanks’ balanced salt solution, trypsinized (0.25% trypsin) for 15 min, triturated, and seeded on poly-D-lysine (50 μg/ml) pre-coated 24-well plates. Cultures were maintained in Neurobasal medium supplemented with 2% v/v B27, 2μM cytosine arabinoside, 1mM pyruvic acid, 2 mM L-glutamine, and penicillin/streptomycin for seven days.

### Direct toxicity of MH towards neuronal cells

NSC-34 murine neuron-like cells (4 x 10^5^ cells/ml) and SH-SY5Y human neuron-like cells (5 x 10^5^ cells/ml) were seeded in 24-well plates in 400 μl and 500 μl, respectively, of DMEM-F12 containing 5% CBS and antibiotics. After 24 h cell culture medium was refreshed, and cells were treated with increasing concentrations (2–50 μg/ml) of MH or with the vehicle solution (deionized H_2_O). Following an incubation period of 2, 24, or 72 h, neuron-like cell viability was assessed by the MTT assay as described below. A similar experiment was also conducted utilizing primary murine cortical neurons that were seeded in 24-well plates at a cell density of 1 x 10^5^ cells/ml in Neurobasal medium and exposed for 24 h to MH or their vehicle solution. In an additional series of experiments TAK-242 (1–20 μM) or its vehicle solution was added to both NSC-34 and SH-SY5Y neuron-like cells 30 min prior to their treatment with 50 μg/ml MH to assess the potential involvement of TLR4 in the cytotoxic actions of extracellular histones.

### Secretion of cytotoxins by BV-2 murine microglia and U118 MG human astrocytic cells

Secretion of cytotoxins by BV-2 murine microglia cells was assessed by transferring their supernatants onto NSC-34 murine neuron-like cells following previously published experimental protocols with minor modifications [48]. BV-2 murine microglia were seeded in 24-well plates at 2 x 10^5^ cells/ml in one ml DMEM-F12 containing 5% CBS and antibiotics. Cells were allowed to adhere for 24 h, then their supernatants were aspirated and replaced with fresh medium. The cells were treated with increasing concentrations of MH or with the vehicle solution (deionized H_2_O) for 15 min, followed by exposure to either LPS (400 ng/ml), murine IFN-γ (150 U/ml), the combination of LPS (20 ng/ml) plus IFN-γ (150 U/ml), or the vehicle solution (PBS). The concentrations of MH used in this study, 2, 10, and 50 μg/ml, were selected based on previous research showing their DAMP-like activity at this range [39]. The concentrations of LPS and IFN-γ were selected based on previous studies that reported either a lack of activation or submaximal activation when these stimulants were administered independently, while their combination induced a significant cytotoxic response by microglial cells [49]. After an additional 24 h incubation period, cell-free supernatants were collected and used to measure nitrite concentrations by the Griess assay. Concentrations of CXCL10 in cell-free supernatants were measured by an ELISA. BV-2 microglia viability was measured using the MTT assay.

To investigate the effect of MH on the secretion of cytotoxins by BV-2 microglia, 300 μl of cell-free supernatants were transferred to separate wells containing NSC-34 murine neuron-like cells that had been seeded 24 h earlier at 4 x 10^5^ cells/ml in 400 μl of DMEM-F12 containing 5% CBS and antibiotics. Additionally, 100 μl of fresh DMEM-F12 containing 10% CBS and antibiotics were added to the wells. After 72 h incubation, NSC-34 neuron-like cell viability was measured using the MTT assay.

To investigate the effect of MH on the secretion of cytotoxins by U118 MG human astrocytic cells, similar experiments were conducted using SH-SY5Y human neuron-like cells as targets [12]. U118 MG cells were seeded in 24-well plates at 2 x 10^5^ cells/ml in one ml DMEM-F12 containing 5% CBS and antibiotics. Cells were allowed to adhere for 24 h and their supernatants replaced with fresh medium. MH or their vehicle solution was added for 15 min, followed by exposure to the established astrocyte stimulants LPS (400 ng/ml), human IFN-γ (100 U/ml), or their vehicle solution (PBS) for 48 h. Subsequently, 400 μl of cell-free supernatants were transferred to separate wells containing SH-SY5Y human neuron-like cells that were seeded 24 h earlier at 6 x 10^5^ cells/ml in 400 μl of DMEM-F12 containing 5% CBS and antibiotics and SH-SY5Y neuron-like cell viability was measured after 72 h incubation.

### Nitric oxide release by BV-2 murine microglia

Concentrations of nitrite, the breakdown product of nitric oxide, were measured by the Griess assay as previously described [50]. 50 μl of the Griess reagent (1% w/v sulfanilamide, 2.5% v/v phosphoric acid, 0.1% w/v *N*-(1-naphthyl)ethylenediamine dihydrochloride in H_2_O) were added to 50 μl of BV-2 cell-free supernatants and optical densities measured at 570 nm using the FLUOstar Omega microplate reader (BMG Biotech, Ortenberg, Germany). Concentrations of nitrite in the cell-free supernatants were interpolated by comparison to samples containing standardized concentrations of nitrite in cell culture medium.

### Glutamate release by BV-2 murine microglia

The concentration of glutamate in cell-free supernatants was quantified by an enzymatic assay as previously described with minor modifications [49, 51]. BV-2 murine microglia were plated and stimulated as described in section 2.3, with the exception that phenol-free DMEM-F12 was used. After 24 h incubation, 20 μl of cell-free supernatants were transferred to 96-well plates and mixed with 155 μl triethanolamine buffer (80 mM triethanolamine, 20 mM potassium phosphate, pH 8.6) containing 12 μl INT, 5.5 μl β-NAD, and 6.2 μl diaphorase. Optical density was measured at 490 nm using the FLUOstar Omega microplate reader and the enzymatic reaction was initiated by adding 25 μl L-glutamic dehydrogenase solution. The final concentrations of reagents were as follows: INT 72 μg/ml; β-NAD 535 μg/ml; diaphorase 0.6 U/ml; L-glutamic dehydrogenase 18.8 U/ml. Optical densities were recorded every five min until the maximum reading was reached. Concentrations of glutamate in the samples were determined using standards containing glutamate in phenol-free DMEM-F12 after subtracting optical density values obtained from control samples containing tissue culture medium only from values obtained from cell supernatants and glutamate standards [52].

### Phagocytic activity of BV-2 murine microglia

Phagocytosis of latex beads by BV-2 murine cells was studied as previously described with minor modifications [27, 50]. BV-2 murine microglia were seeded at a density of 5 x 10^4^ cells/ml in 500 μl of DMEM-F12 containing 5% CBS and antibiotics in four-chambered glass-bottom Petri dishes. Cells were allowed to adhere for 24 h followed by treatment with MH (50 μg/ml), LPS (400 ng/ml), or their vehicle solutions for 24 h. Cell culture medium was replaced, three μl of fluorescent latex beads (one μm diameter, 10 mg/ml initial concentration) were added, and cell cultures incubated for one h. External beads were washed away with PBS, and cells were fixed by adding 500 μl of cold 70% ethanol for five min. Prior to imaging, ethanol was replaced with 500 μl of PBS, and five μL bisbenzimide (2 μg/ml) nuclear stain was added to each chamber. Cells were imaged with a Zeiss AxioObserver.Z1 inverted widefield fluorescence microscope. Zen 2.0 image acquisition software was used to measure fluorescence at an excitation/emission of 365/445 nm for bisbenzimide and 474/537 nm for the fluorescent beads. Corrected total cell fluorescence values were measured by an independent investigator blinded to the experimental condition using the National Institutes of Health (NIH) ImageJ (FIJI build, imagej.net).

### Assessing cell viability

Cell viability was monitored by an assay that measured the reduction of MTT to insoluble purple formazan crystals by viable cells [53]. A previously described procedure was used with minor modifications [12]. Cells were incubated with MTT (0.5 mg/ml) at 37 ˚C for one h. Subsequently, formazan crystals were dissolved by adding a volume of sodium lauryl sulfate (20% w/v)/*N*,*N*-dimethylformamide (50% v/v) solution equal to that of the culture medium present in the well, then shaking the plates for three h. Optical densities were measured at 570 nm using the FLUOstar Omega microplate reader. Cell viability data are presented as a percentage compared to values obtained from cells incubated in growth medium alone.

### Statistical analysis

Data were analyzed using randomized block design one-way analysis of variance (ANOVA), followed by either Dunnett’s or Tukey’s post hoc test. Independent experiments were performed on separate days and data are presented as means ± standard error of the mean (SEM). Statistical significance was established as P < 0.05. GraphPad Prism (version 8.0.1, GraphPad Software Inc., La Jolla, CA, USA) was used to perform all statistical analyses and to construct all graphs.

## Results

Previous studies show that exposure of neuronal cell lines and cultured primary rat neurons to extracellular histones causes toxic effects that differ between histone isoforms. For example, Gilthorpe et al. [44] report that H1 is toxic towards cultured rat cortical neurons after 24 h exposure, while treatment with either H2A, H2B, H3, or H4 does not induce toxicity. Studies with non-CNS cell types have recorded direct toxicity of extracellular histones after 1–24 h exposure times [38, 54]. We assessed the effects of MH on NSC-34 murine and SH-SY5Y human neuron-like cells after 2, 24, and 72 h incubation. In addition, we studied the effects of MH on primary murine neurons after a 24 h incubation period.

**Fig 1:**
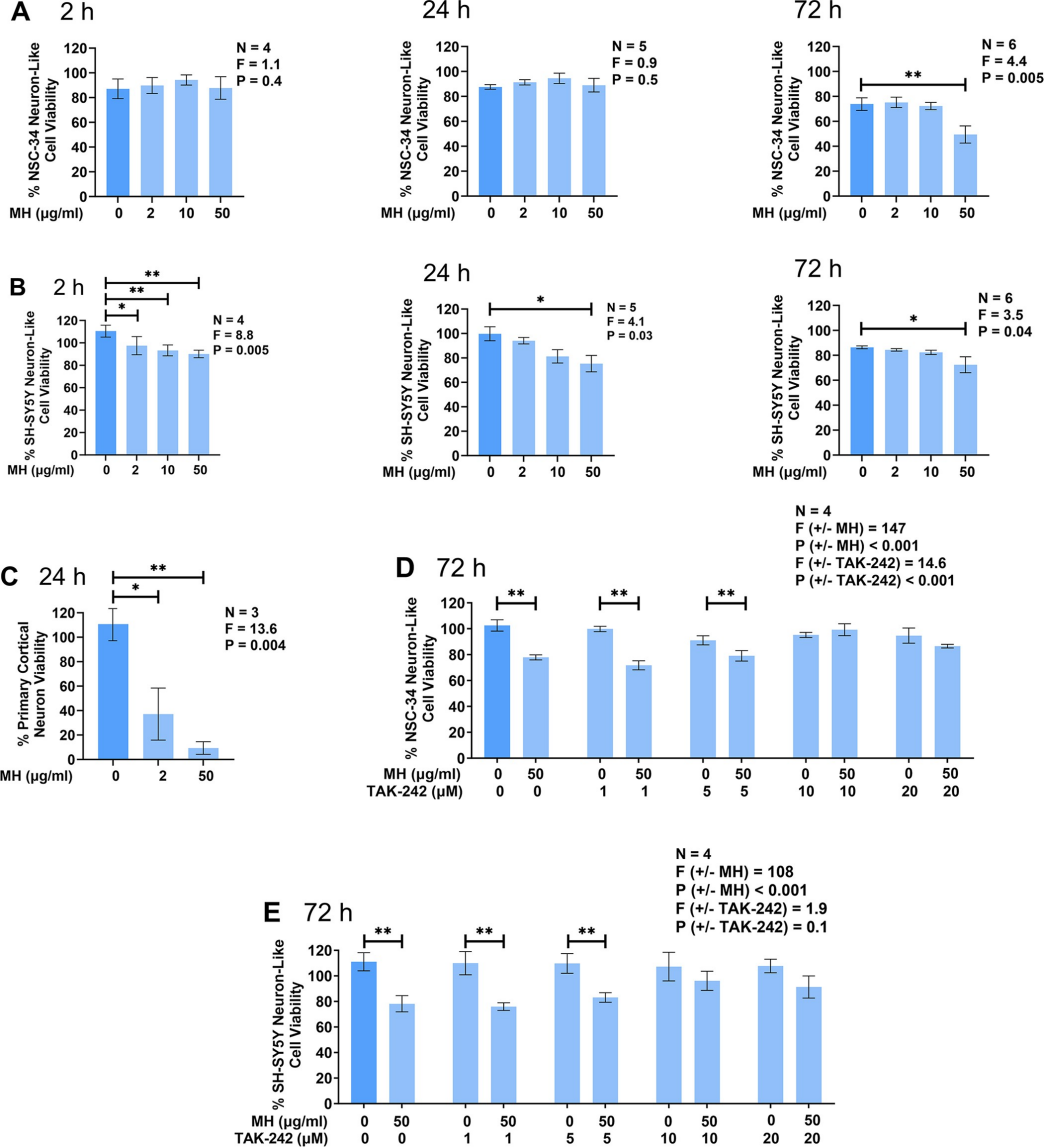
Extracellular MH reduce neuronal cell viability. Effects of MH on the viability of cultured NSC-34 murine neuron-like cells (A,D), SH-SY5Y human neuron-like cells (B,E), and primary murine neurons (C). Following 2, 24, or 72 h incubation with MH (2–50 μg/ml) neuronal cell viability was measured by the MTT assay. In two of the experiments TAK-242 (0, 1, 5, 10, and 20 μM) was added to NSC-34 (D) or SH-SY5Y (E) neuron-like cells 30 min prior to their treatment with 50 μg/ml MH or its vehicle solution. Data (means ± SEM) from three to six independent experiments performed on separate days are presented. * P < 0.05 and ** P < 0.01, according to Dunnett’s (A-C) or Tukey’s (D,E) post-hoc test. P and F values for the one-way (A-C) or two-way randomized block (D,E) ANOVA are displayed.

Fig 1A demonstrates that extracellular MH at 50 μg/ml caused an approximate 25% reduction in viability of NSC-34 murine neuron-like cells after 72 h incubation; however, shorter incubation periods or lower concentrations of MH did not lead to its cytotoxicity towards this cell type. The direct cytotoxic effect of MH towards SH-SY5Y human neuron-like cells was more rapid, where exposure to MH at 50 μg/ml caused an approximate 20% reduction in viability after 2, 24, and 72 h incubation (Fig 1B). In addition, MH at both 2 and 10 μg/ml induced a marginal, yet statistically significant, decrease in SH-SY5Y human neuron-like cell viability after a 2 h incubation period (Fig 1B). Furthermore, treatment of murine cortical neurons with MH at 50 μg/ml resulted in nearly a 90% decrease in viability, while 2 μg/ml caused more than a 50% reduction in viability after 24 h incubation (Fig 1C).

Since previous studies indicate that extracellular histones signal through interactions with TLR4 [55, 56], we hypothesized that this receptor was mediating interactions between MH and the two types of neuron-like cells used. To test this hypothesis, NSC-34 cells (Fig 1D) and SH-SY5Y cells (Fig 1E) were treated with increasing concentrations of TAK-242 prior to exposure to MH. TAK-242 at the two lowest concentrations used in this study (1 and 5 μM) failed to inhibit the cytotoxic actions of extracellular MH on both neuron-like cell types; however, in the presence of the two highest concentrations of TAK-242 (10 and 20 μM) MH were unable to induce significant death of neuron-like cells indicating possible involvement of TLR4 in MH-mediated cytotoxicity.

**Fig 2:**
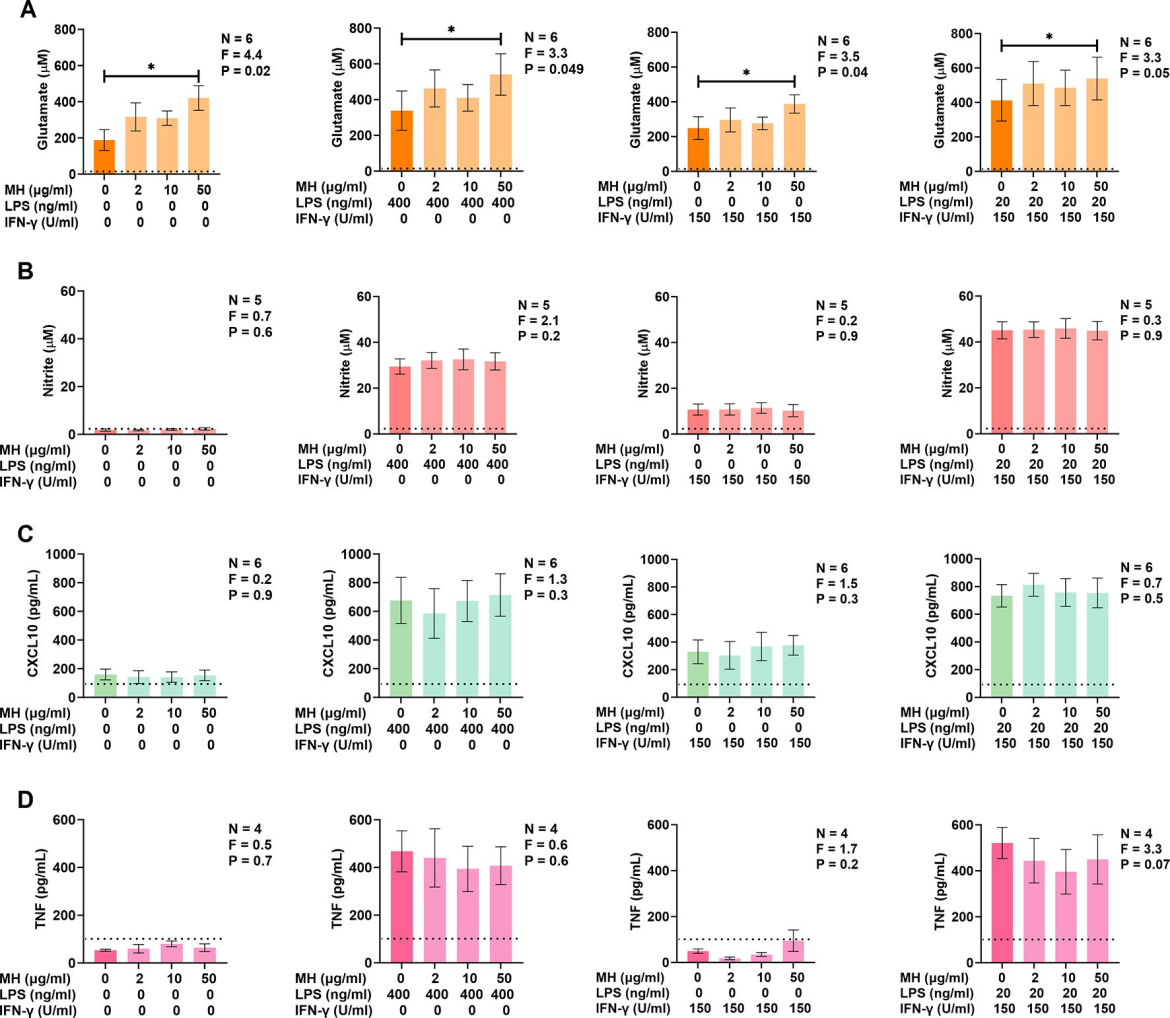
Effects of extracellular MH on the secretion of pro-inflammatory and potentially neurotoxic molecules by BV-2 murine microglia. Effects of MH on the secretion of glutamate (A), NO (B), CXCL10 (C), and TNF (D) by unstimulated and stimulated BV-2 murine microglia. Cells were exposed to MH (2–50 μg/ml) on their own, or cells were also stimulated 15 min later with either LPS (400 ng/ml), IFN-γ (150 U/ml), or a combination of LPS (20 ng/ml) plus IFN-γ (150 U/ml). Following a 24 h incubation period, concentrations of glutamate in cell-free supernatants were measured by an enzyme-based assay (A); concentrations of nitrite in cell-free supernatants were measured by the Griess assay (B); and concentrations of CXCL10 (C) and TNF (D) were measured by ELISAs. Data (means ± SEM) from four to six independent experiments performed on separate days are presented. * P < 0.05 according to Dunnett’s post-hoc test. P and F values for the one-way randomized block ANOVA are displayed, and the detection limit of each assay is indicated by a dotted line.

Previous studies demonstrate the ability of MH to modify the pro-inflammatory responses of several types of peripheral mononuclear cells [38, 39]. Therefore, we investigated whether MH could induce or regulate the inflammatory response of unstimulated and stimulated BV-2 murine microglia by examining the effects of MH on the secretion of four pro-inflammatory and potentially neurotoxic molecules: glutamate, NO, CXCL10, and TNF. We utilized the bacterial endotoxin LPS and the cytokine IFN-γ, which activate TLR4 and the IFN-γ receptor (IFNGR), respectively, as established pro-inflammatory stimulants of microglia [57, 58]. Fig 2A demonstrates that MH at 50 μg/ml induced the secretion of the excitotoxic neurotransmitter, glutamate, by BV-2 murine microglia. Furthermore, MH at the same concentration significantly upregulated the secretion of glutamate by BV-2 cells when added as a co-stimulant to LPS, IFN-γ, or a combination of LPS plus IFN-γ. MH at the concentrations tested (2–50 μg/ml) did not modulate secretion of NO (Fig 2B), CXCL10 (Fig 2C), or TNF (Fig 2D) by unstimulated or stimulated BV-2 murine microglia.

**Fig 3:**
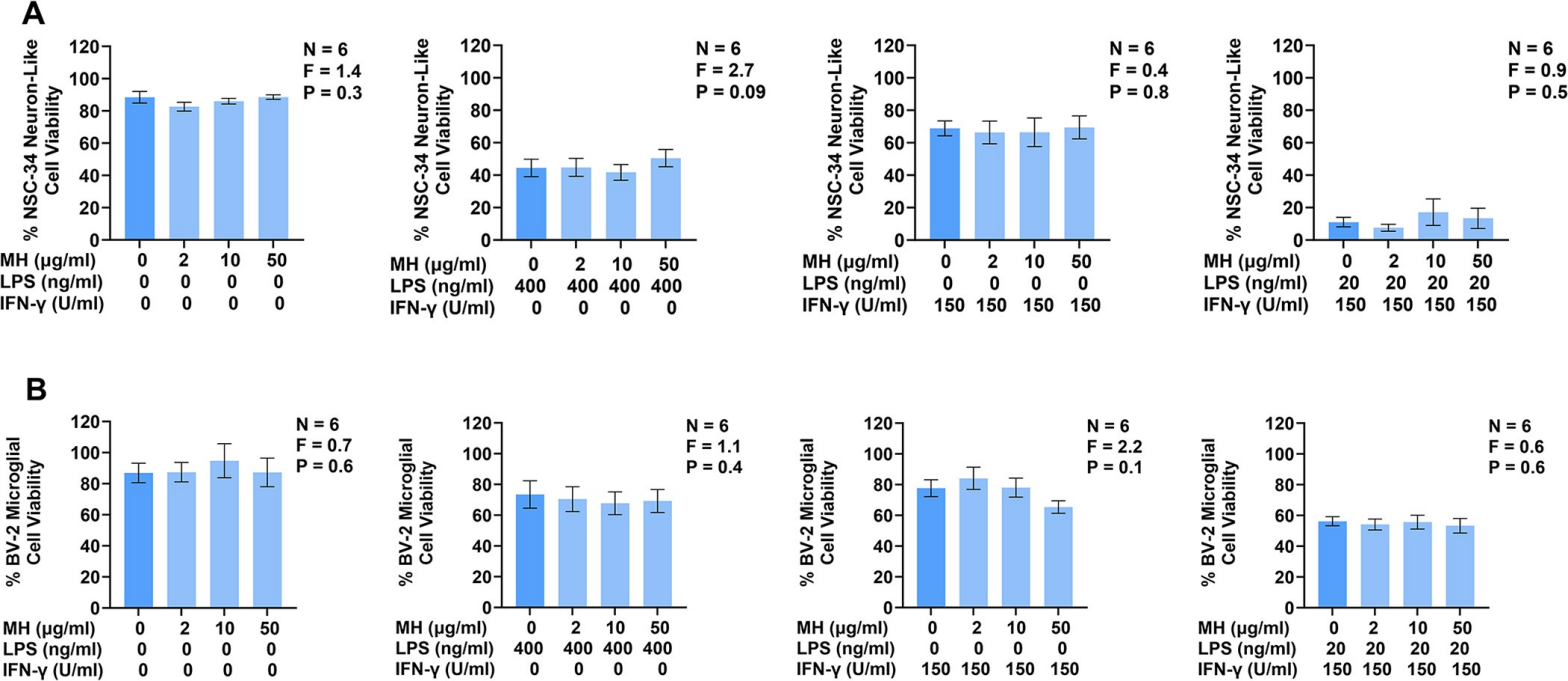
Extracellular MH do not induce BV-2 murine microglia cytotoxicity towards NSC-34 murine neuron-like cells, or modulate the cytotoxicity of immune-stimulated BV-2 murine microglia towards NSC-34 cells. Effects of MH on BV-2 microglia-mediated cytotoxicity towards NSC-34 murine neuron-like cells (A) and viability of unstimulated and stimulated BV-2 murine microglia (B). BV-2 cells were exposed to MH (2–50 μg/ml) on their own, or cells were also stimulated 15 min later with either LPS (400 ng/ml), IFN-γ (150 U/ml), or a combination of LPS (20 ng/ml) plus IFN-γ (150 U/ml). Following a 24 h incubation period, supernatants from BV-2 cells were transferred onto NSC-34 cells and the viability of BV-2 cells was measured using the MTT assay (B). 72 h later, the viability of NSC-34 cells was measured using the MTT assay (A). Data (means ± SEM) from six independent experiments performed on separate days are presented. P and F values for the one-way randomized blocks ANOVA are displayed.

Since MH induced the release of glutamate, which is an established neurotoxin [59], we studied if MH, alone or in combination with pro-inflammatory stimulants, could induce or modulate toxicity of BV-2 murine microglia towards NSC-34 murine neuron-like cells. Our previous studies demonstrate that certain combinations of inflammatory mediators and cytokines, such as LPS in combination with IFN-γ, and IL-6 in combination with TNF, induce microglia-mediated toxicity towards neurons [49]. Thus, we utilized both LPS and IFN-γ as stimulants to investigate whether MH modulate the toxicity of BV-2 murine microglia towards NSC-34 murine neuron-like cells. Fig 3A illustrates that the supernatants from unstimulated BV-2 microglia, or cells exposed to MH alone, were not toxic towards NSC-34 cells resulting in ~90% cell viability after 72 h incubation in the supernatants from unstimulated BV-2 cells when compared to the exposure to cell culture medium only (Fig 3A, left panel). Consistent with previous studies, incubation of NSC-34 neuron-like cells in supernatants from immune-stimulated BV-2 murine microglia led to a reduction in their viability [48]. The NSC-34 cell viability decreased to ~45% after incubation in supernatants from LPS-stimulated BV-2 cells, with the corresponding viability values being ~70% and ~ 10% when IFN-γ and LPS plus IFN-γ respectively were used as stimulants (Fig 3A, panels left to right). Treatment of stimulated BV-2 cells with MH (2–50 μg/ml) neither decreased nor potentiated the cytotoxicity of their supernatants after 72 h incubation. Fig 3B illustrates that MH alone did not alter the viability of BV-2 cells. MH also had no additional effect on the viability of BV-2 cells stimulated with LPS, IFN-γ, or LPS plus IFN-γ compared to cells treated with the respective pro-inflammatory stimulants only.

**Fig 4:**
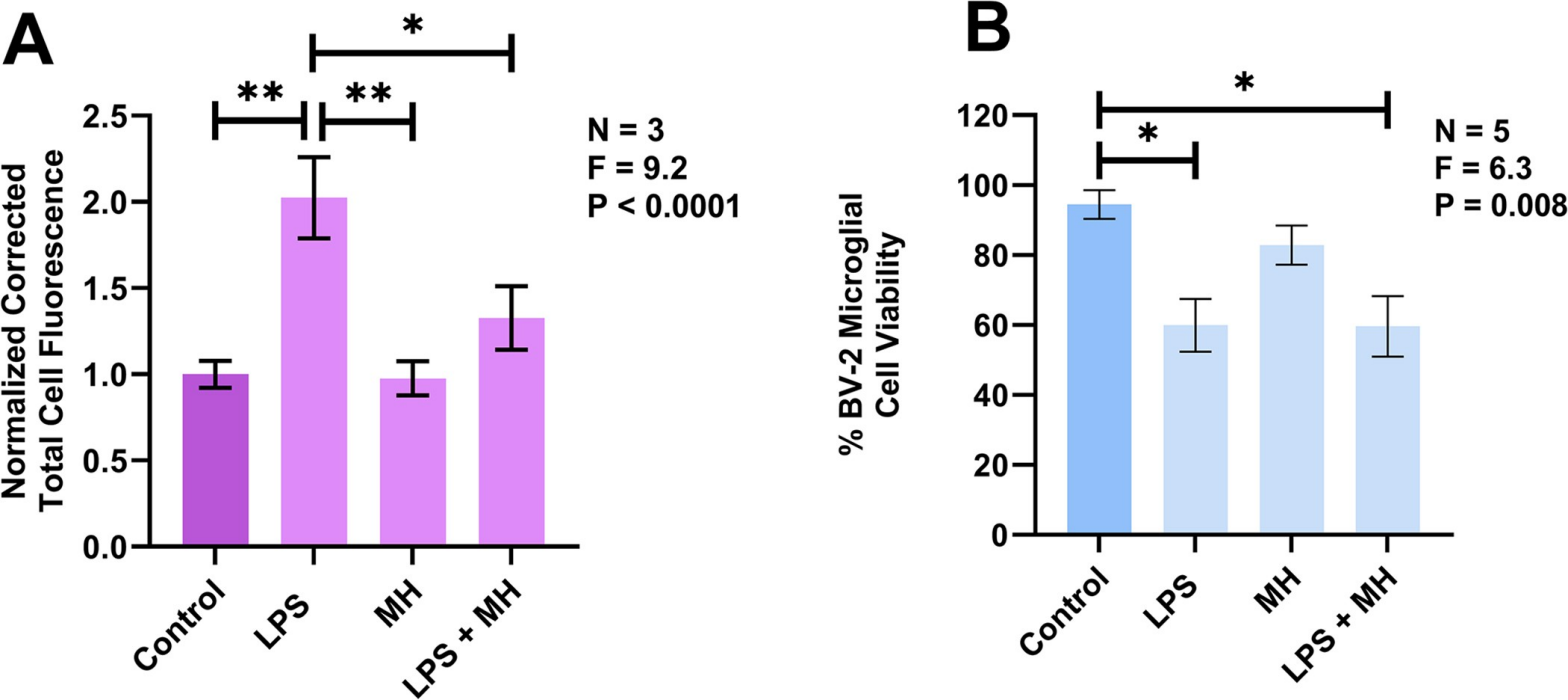
Extracellular MH downregulate LPS-induced phagocytic activity of BV-2 murine microglia. Effects of LPS and MH on phagocytosis of latex beads by BV-2 murine microglia (A) and their viability (B). Cells were incubated with LPS (400 ng/ml), MH (50 μg/ml), a combination of LPS (400 ng/ml) and MH (50 μg/ml), or their vehicle solutions for 24 h, followed by exposure to fluorescent latex beads for one h. Corrected total cell fluorescence values (means ± SEM) from 125–147 randomly selected cells from three independent experiments were measured and normalized against values obtained from control samples exposed to vehicle solutions only. Statistical analyses were performed before these data transformations. Viability data (means ± SEM) from three or five independent experiments performed on separate days are presented. * P < 0.05 and ** P < 0.01, according to Tukey’s post-hoc tests. P and F values for the one-way randomized block ANOVA are displayed.

To the best of our knowledge, regulation of microglia phagocytic activity by extracellular histones has not been reported. Thus, we investigated the effects of MH on BV-2 murine microglia phagocytosis of latex beads. LPS is well known for its ability to upregulate phagocytic activity in multiple cell types, including microglia; therefore, we used it as an established inducer of BV-2 murine microglia phagocytic activity [60, 61]. Fig 4A illustrates that incubation of BV-2 cells with LPS for 24 h significantly upregulated their uptake of fluorescent latex beads. In the absence of this pro-inflammatory stimulant, 24 h exposure of BV-2 cells to MH at 50 μg/ml did not alter their uptake of fluorescent latex beads compared to vehicle-treated control cells. BV-2 cells that were stimulated with LPS in the presence of MH at 50 μg/ml for 24 h had significantly reduced uptake of latex beads compared to cells treated with LPS alone. It is important to note that MH alone at 50 μg/ml did not reduce the viability of BV-2 cells (Fig 4B). Co-exposure of cells to LPS and MH at 50 μg/ml did not reduce their viability compared to cells treated with LPS alone, which on its own induced significant cytotoxicity compared to unstimulated cells.

**Fig 5:**
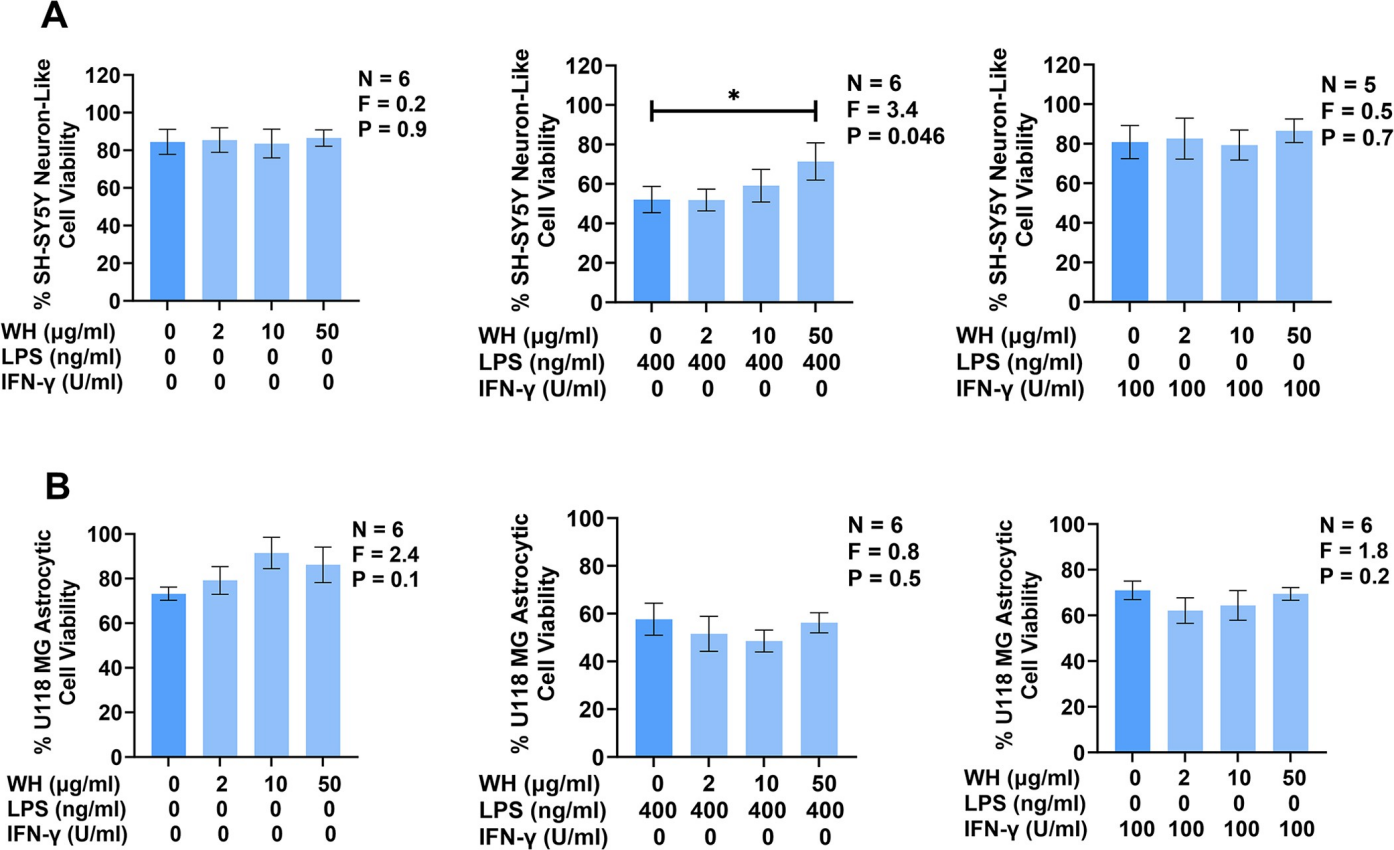
Extracellular MH downregulate LPS-stimulated U118 MG human astrocytic cell mediated toxicity towards SH-SY5Y human neuron-like cells and are not cytotoxic towards U118 MG cells.

The immune responses of other CNS glial cells, such as astrocytes, are increasingly recognized as having a critical role in the pathogenesis of several neurodegenerative diseases [62]. Therefore, we investigated whether MH could induce or modulate toxicity of U118 MG human astrocytic cells towards SH-SY5Y human neuron-like cells. MH were added to U118 MG cells that were then either left unstimulated or stimulated with LPS or IFN-γ, which are known to induce toxicity of human astrocytic cells towards SH-SY5Y human neuron-like cells [62, 63]. Fig 5A illustrates that treatment of U118 MG human astrocytic cells with MH in the absence of pro-inflammatory stimulants did not induce the cytotoxicity of this cell type towards SH-SY5Y human neuron-like cells viability of which remained at ~85% (Fig 5A, left panel). Incubation of SH-SY5Y cells in supernatants from U118 MG cells that had been stimulated with LPS reduced viability of SH-SY5Y cells to ~50% (Fig 5A). Treatment of LPS-stimulated U118 MG cells with MH significantly decreased their cytotoxicity towards SH-SY5Y cells compared to U118 MG cells stimulated with LPS in the absence of MH resulting in SH-SY5Y cell viability restored to ~70% in the presence of 50 μg/ml MH. Exposure to MH (2–50 μg/ml) neither inhibited nor potentiated the cytotoxicity of U118 MG human astrocytic cells stimulated with IFN-γ. Fig 5B illustrates that MH alone did not alter the viability of U118 MG cells. Additionally, MH had no effect on the viability of U118 MG cells stimulated with LPS or IFN-γ compared to cells treated with the respective pro-inflammatory stimulant only.

Effects of MH on U118 MG astrocytic cell-mediated cytotoxicity towards SH-SY5Y human neuron-like cells and viability of unstimulated and stimulated U118 MG human astrocytic cells. U118 MG cells were exposed to MH (2–50 μg/ml) on their own, or cells were also stimulated 15 min later with either LPS (400 ng/ml) or IFN-γ (100 U/ml). Following a 48 h incubation period, supernatants from U118 MG cells were transferred onto SH-SY5Y cells and the viability of U118 MG cells was measured using the MTT assay. 72 h later, the viability of SH-SY5Y cells was measured using the MTT assay. Data (means ± SEM) from five to six independent experiments performed on separate days are presented. * P < 0.05 according to Dunnett’s post-hoc test. P and F values for the one-way randomized blocks ANOVA are displayed.

## Discussion

Extracellular histones are widely recognized for their pathophysiological contributions to many inflammatory conditions of the periphery, including traumatic injury, sepsis, and organ failure [40, 64, 65]. The 2–50 μg/ml concentration range of extracellular histones used in our experiments has been utilized routinely in studies assessing their effects in peripheral tissues [38, 39]. In addition, Alhamdi et al. [66] demonstrate that histone concentrations could reach 150 μg/ml in sera of some patients with sepsis and left ventricular dysfunction. To the best of our knowledge, only two studies have measured concentrations of extracellular histones in the CNS [67, 68]. Thus, Schutzer et al. [67] demonstrate that the extracellular histones H1, H2A, H2B, H3, and H4 are present at detectable levels in human cerebrospinal fluid (CSF), and Begcevic et al. [68] report 1.6 times higher concentrations of H2A in the CSF of AD patients compared to age-matched controls. However, the experimental techniques utilized by these studies lack the ability to differentiate between the distinct isoforms of histones and their mixture. Histones can enter the brain parenchyma through a damaged blood-brain barrier (BBB), which is a hallmark of neuroinflammatory and neurodegenerative diseases [69]. Notably, Villalba et al. [70] report that MH themselves are capable of causing the breakdown of the tight junction proteins in murine brain microvascular endothelial cell monolayers, which strongly indicates their ability to permeabilize the BBB and enter the CNS. These observations indicate that extracellular histone concentrations in the brain parenchyma and sera could be similar under pathological conditions. Thus, the 2–50 μg/ml range of MH concentrations used in this study could be relevant to the pathological states of periphery and CNS.

Numerous studies have documented the direct toxicity of extracellular histones towards several cell types of the periphery, such as human pulmonary microvascular endothelial cells and human vascular smooth muscle cells [64, 71]. Extracellular histones also demonstrate toxic effects *in vivo* where they have been shown to damage murine hepatic and pulmonary tissues [72]. In our cell culture systems extracellular MH (up to 50 μg/ml) were not toxic to either resting or immune-stimulated BV-2 murine microglia or U118 MG human astrocytic cells; however, MH exhibited toxicity towards two different neuron-like cell lines, as well as cultured primary murine cortical neurons.

Previous studies have yielded conflicting results regarding the effects of extracellular histones on neurons. Mishra et al. [46] show that independent administration of all histone isoforms, except H4, triggers neuritogenesis, the development and growth of cellular projections, in cultured primary murine cerebellar neurons, suggesting that extracellular histones have beneficial effects on this cell type [73]. In contrast, H1 induces axonal injury in cultured primary murine neurons [44], and MH are directly toxic to PC12 rat neuron-like cells [74]. Our results support the latter two studies by demonstrating that MH, when applied extracellularly, significantly reduced the viability of three different types of neuronal cells: primary murine neurons, NSC-34 murine neuron-like cells, and SH-SY5Y human neuron-like cells. It is important to note that the neuron-like cells as well as the cultured murine primary neurons used in this study differ significantly from human neurons and therefore do not provide comprehensive neuronal models that would be directly relevant to human neurodegenerative diseases. Human induced pluripotent stem cell (iPSC)-derived neuronal cultures and brain organoids could be used in future studies to determine effects of extracellular histones on various types of human neurons.

MH were more efficacious towards primary murine neurons where they induced an approximate 90% reduction in viability after 24 h, whereas there was no reduction or an approximate 20% reduction in the viability of NSC-34 murine and SH-SY5Y cells, respectively, at this time point. Moreover, the toxic activity of MH was more potent towards primary neurons, which were affected by MH at 2 μg/ml after 24 h incubation, compared to NSC-34 murine cells, which had no reduction in viability after the same incubation period at all concentrations tested (0–50 μg/ml). Our data corroborate previous studies by Akhter et al. [75] and Nakamura et al. [76] who report that primary murine neurons are more susceptible to insults compared to immortalized murine cell lines due to the differences in their metabolism and cell signaling machinery. For example, TLR4, an established molecular target of extracellular histones, is undetectable in untreated NSC-34 neuron-like cells [77] but is highly expressed in murine primary neurons [78]. Our observations demonstrating direct neurotoxic effects of MH may support the hypothesis that extracellular histones contribute to neuronal death in AD and other neurodegenerative diseases [37, 79]; however, since our studies used undifferentiated neuron-like cell lines and cultured murine neurons, this hypothesis will require further experimental and clinical proof. Nevertheless, inhibiting the neuronal receptors that are used by extracellular histones to induce neurotoxicity could be considered as a candidate therapeutic strategy in neurodegenerative diseases.

Since previous research implicates TLR4 as one of the predominant mediators of the interactions between extracellular histones and diverse cell types [40, 80], we tested the hypothesis that this receptor was responsible for the cytotoxic effects of MH on NSC-34 murine and SH-SY5Y human neuron-like cells. TAK-242, a specific inhibitor of TLR4, at the two highest concentrations eliminated the cytotoxic effect of MH on both neuron-like cell types used in this study supporting TLR4 as one of the candidate receptors mediating the neurotoxic effects of extracellular histones. Further research is needed to elucidate other possible molecular interactions that underlie the observed effects of MH on neuronal cells as well as the intracellular signaling pathways engaged by histone-neuron interactions.

To the best of our knowledge, only three studies have reported the immunomodulatory actions of extracellular histones on microglia model cells with the majority of these observations made by using individual histone isoforms instead of MH. Gilthorpe et al. [44] observe upregulated chemotaxis and expression of the activation marker major histocompatibility (MHC) class II by primary murine microglia in the presence of extracellular H1. Munemasa [43] demonstrates in a murine model of primary acute-closure glaucoma that extracellular H2B induces retinal ganglion degeneration, which is presumably mediated by activation of microglia. To date, the only study to document the pro-inflammatory activity of extracellular histones on human microglia-like cells is by Westman et al. [39], who utilize THP-1 human monocytic cells, a well-established model of human microglia [81], to demonstrate that treatment with either MH or H4 upregulates production of CXCL10 and TNF by this cell type.

We quantified the secretion of four pro-inflammatory and potentially neurotoxic molecules by unstimulated and immune-activated BV-2 murine microglia to determine if they were differentially regulated by extracellular MH. Even though secretion of the excitotoxic neurotransmitter glutamate was upregulated in the presence of MH, these proteins failed to modulate the secretion of NO, CXCL10, or TNF by BV-2 murine microglia. Despite eliciting secretion of the neurotoxin glutamate, MH did not induce overall cytotoxicity of BV-2 murine microglia towards NSC-34 murine neuron-like cells. Bernath et al. [49] report a similar result for ATP, a well-established mitochondrial DAMP, which upregulates the secretion of glutamate, but not CXCL10 or TNF, by BV-2 murine microglia and does not induce their toxicity towards NSC-34 murine neuron-like cells.

Microglial phagocytic activity plays a protective role in the brain by enabling the removal of pathogens, misfolded proteins, and cell debris which ultimately aids in the resolution of neuroinflammation [82]. This function is impaired during aging and many CNS pathologies [83, 84]. We demonstrated that MH inhibited LPS-induced phagocytic activity of BV-2 murine microglia while having no effect on the basal phagocytic rate of these cells. This observation may indicate another DAMP-like effect of MH on microglia, in addition to inducing the release of glutamate by this cell type. Our findings align with prior research that demonstrates the effects of extracellular histones on the phagocytic activity of non-CNS phagocytes. For example, Friggeri et al. [85] report that H3 and H4 downregulate the phagocytosis of neutrophils and thymocytes by murine peritoneal macrophages.

Several DAMPs are known to affect specific immune functions of astrocytes and induce their toxicity towards neuronal cells. For example, the mitochondrial DAMP cytochrome C induces TLR4-dependent secretion of the pro-inflammatory IL-1β by primary human astrocytes, as well as the toxicity of U118 MG human astrocytic cells towards SH-SY5Y human neuron-like cells [12]. We observed that MH inhibited the cytotoxic potential of LPS-stimulated U118 MG cells, which contrasts the pro-inflammatory effects of MH on microglia described above. A possible explanation for this result could be that MH may have outcompeted LPS for interactions with TLR4. Previous studies show that LPS induces the inflammatory responses of astrocytes in a TLR4-dependent manner [86], and that TLR4 is also engaged by MH [55, 80]. Given that MH were administered at a higher concentration (50 μg/ml) than LPS (0.5 μg/ml), it is possible that MH prevented LPS from binding to TLR4, thereby attenuating the LPS-induced toxicity of astrocytic cells towards neuron-like cells. This hypothesis is supported by microscale thermophoresis data showing that the dissociation constants of H4 and LPS for TLR4 are very similar [87, 88]. Cell line-specific effects of MH also cannot be ruled out since, for example, our observed effectiveness of LPS as an inducer of U118 MG astrocytic cell toxicity towards SH-SY5Y neuron-like cells differs from a lack of such an effect reported by Hashioka et al. [63] using human U373 MG astrocytic cells.

Our data showing potentially pro-inflammatory effects of MH on microglia, and their protective activity on astrocytic cells do not provide conclusive support to the hypothesis that extracellular histones contribute to the pathogenesis of AD due to their pro-inflammatory activity [38]. Our observations indicate that the neuroimmunomodulatory effects of extracellular histones could be glial cell type specific, and also depend on the immune stimuli encountered by these cells. Further research using primary human and other animal microglia and astrocytes as well as human iPSC-derived glia should be conducted to determine species-specific effects of extracellular histones on glial cells. Future studies should also use individual histone subtypes, alone or in combination, which could reveal differential activity of histone isoforms towards various CNS cell types. Advanced molecular biology and proteomics techniques could be applied to elucidate the changes in glial intracellular signaling networks induced by their interaction with extracellular histones under different immunostimulatory conditions.

## Supporting information

S1 Table Raw data. (XLSX)
